# Fish Allergenicity Modulation Using Tailored Enriched Diets—Where Are We?

**DOI:** 10.3389/fphys.2022.897168

**Published:** 2022-05-25

**Authors:** Denise Schrama, Rebecca Czolk, Cláudia Raposo de Magalhães, Annette Kuehn, Pedro M. Rodrigues

**Affiliations:** ^1^ Centre of Marine Sciences (CCMAR), Universidade do Algarve, Faro, Portugal; ^2^ Universidade do Algarve, Faro, Portugal; ^3^ Department of Infection and Immunity, Luxembourg Institute of Health, Esch-sur-Alzette, Luxembourg; ^4^ Faculty of Science, Technology and Medicine, University of Luxembourg, Esch-sur-Alzette, Luxembourg

**Keywords:** fish allergies, fish nutrition, parvalbumin, creatine, EDTA

## Abstract

Food allergy is an abnormal immune response to specific proteins in a certain food. The chronicity, prevalence, and the potential fatality of food allergy, make it a serious socio-economic problem. Fish is considered the third most allergenic food in the world, affecting part of the world population with a higher incidence in children and adolescents. The main allergen in fish, responsible for the large majority of fish-allergic reactions in sensitized patients, is a small and stable calcium-binding muscle protein named beta-parvalbumin. Targeting the expression or/and the 3D conformation of this protein by adding specific molecules to fish diets has been the innovative strategy of some researchers in the fields of fish allergies and nutrition. This has shown promising results, namely when the apo-form of *β*-parvalbumin is induced, leading in the case of gilthead seabream to a 50% reduction of IgE-reactivity in fish allergic patients.

## Introduction

**FIGURE 1:**
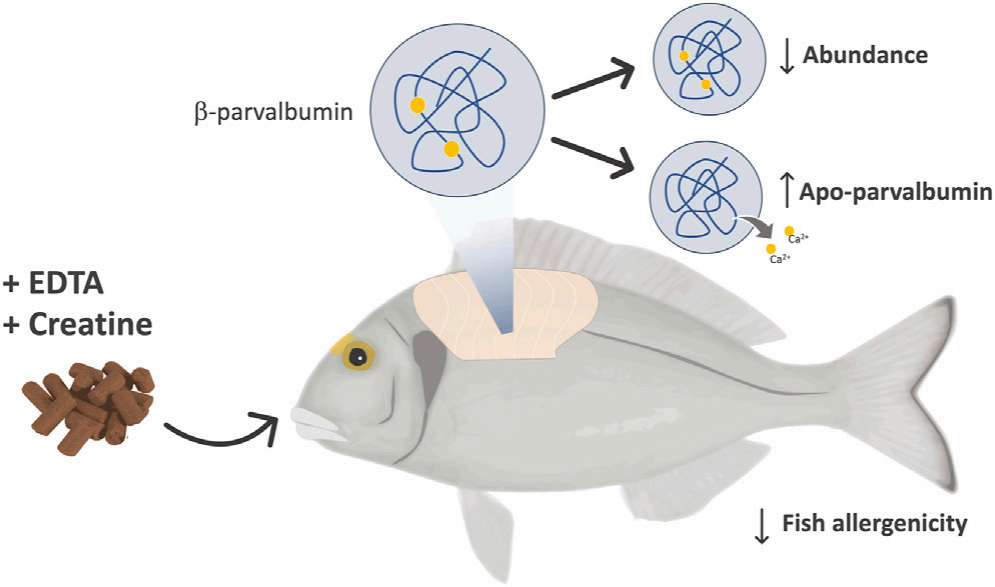
From diagnosis to traditional and advanced clinical approaches to manage fish-allergic patients. Under strict avoidance of any type of fish, patients risk maintaining their food allergy over lifetime. The consumption of single, individually tolerated fish species contributes to a balanced diet and most likely, promotes tolerance development. Hypoallergenic fish species, either naturally occurring or produced in targeted aquaculture, are key components to implement such a personalized approach in patient care.

The World Health Organization considers fish as one of the most frequent causes of food allergies. The prevalence of fish allergy is especially high in the young population, reaching up to 1% based on oral food challenge data and up to 7% based on questionnaires (Moonesinghe et al., 2016; Klueber et al., 2019). Allergy rates correlate also with a pronounced exposure to fish, either by consumption in local diets along coastal lines or by fish handling in the occupational field of fish-processing production lines (<36%) (Bonlokke et al., 2019). Allergic patients experience from mild and local over to severe and systemic reactions upon ingestion, inhalation, or skin contact with fish. The ingestion of fish in the low milligram range is sufficient to provoke clinical symptoms (e.g. 27.3 mg determined as ED10 value for cod) (Ballmer-Weber et al., 2015; Sørensen et al., 2017; Houben et al., 2020). Fish allergy often persists over a lifetime. Natural tolerance to fish might develop until adolescence, especially in patients with a disease onset during their first years of life (Xepapadaki et al., 2021). The diagnosis of fish allergy is established by combining the medical history with results from serological specific IgE-antibody determination and results from skin reactivity tests using authentic fish or fish extracts (Matricardi et al., 2016; Hilger et al., 2017; Klueber et al., 2019; Buyuktiryaki et al., 2021). Oral food challenges, i.e. medically supervised intakes of gradually increasing fish doses, are only performed in exceptional cases where the clinical reactivity is questioned (Matricardi et al., 2016). The clinical management of fish-allergic patients refers mainly to a strict avoidance of all kinds of fish (Figure 1). When accidental consumption occurs, allergic symptoms are treated with rescue medication, including antihistamines, *β*-2 sympathomimetic, inhaled corticosteroids and in severe cases, adrenaline (Tedner et al., 2022). Often, fish-allergic patients demand to consume this food because of its nutritional benefits on health, such as via minerals, vitamins, and polyunsaturated fatty acids (Kalic et al., 2021). Fish is an ideal source of omega-3 fatty acids, including EPA (eicosapentaenoic acid) and DHA (docosahexaenoic acid), which are associated with a reduced risk for chronic inflammatory diseases (Maulu et al., 2021; Chen et al., 2022). Dietary recommendations comprise the consumption of up to two servings of fish per week, a consumption level which is associated with a reduced risk for coronary heart disease (EFSA Scientific Committee, 2015; Chen et al., 2022). The diet-related benefit appears even larger than thought. Recent studies demonstrated that the human gut microbiome is influenced positively through fish consumption (Chen et al., 2022). Regular fish intake correlates with a healthy microbiota composition, meaning with an increased bacterial richness and alpha-diversity, which in turn is known to be associated with an improved immune response and a reduced inflammatory disease burden (Dale et al., 2019; Simione et al., 2020). There is a general notion that the traditional way of clinical management might be further advanced towards a more personalized dietary recommendation regarding (Klueber et al., 2019; Dijkema et al., 2020) which fish species the patient might need to avoid and which fish species the patient might consume safely (Figure 1). The benefit of including certain fishes into the nutrition plan of fish-allergic patients is two-fold. First, to balance the food intake via a high-quality and healthy protein source and second, to create opportunities to the immune system for potential tolerance development through targeted exposure (Maulu et al., 2021; Chen et al., 2022). Important for those approaches is the prior knowledge on fish species that are tolerated individually by the patient, a decision procedure that needs to be followed up specifically in the frame of the clinical diagnosis (Matricardi et al., 2016). The level of serum IgE-antibodies directed against fish proteins might be supportive in this decision (Kuehn et al., 2013; Hilger et al., 2017; Kalic et al., 2019; Rubin et al., 2020). A food challenge-based study demonstrated that patients with specific IgE to cod and salmon extract, IgE-levels >8.2 kU_A_/L and >5 kU_A_/L, respectively, shall be advised to avoid fish in general (Sørensen et al., 2017). This is, however, an approach that has the potential to be further advanced into a less stringent and patient-oriented praxis, based on clinical and molecular allergen knowledge (Klueber et al., 2019; Dijkema et al., 2020). Some fish species are better tolerated than others, which might be explained by low fish allergen contents as well as by specific properties of the allergenic fraction (Kuehn et al., 2010; Mourad and Bahna, 2015; Kobayashi et al., 2016b; Kalic et al., 2019; Pérez-Tavarez et al., 2021). Known low allergen content fishes are perch-like fishes, such as tuna and swordfish (Kuehn et al., 2010; Kobayashi Y. et al., 2016). Another fish with reduced molecular allergenicity is ray, a cartilaginous species (Kalic et al., 2019). Those species might be considered as naturally occurring hypoallergenic fish and thus, a valuable alternative to balance the avoidance diet of many fish-allergic patients. To identify, or even create, hypoallergenic fish is considered an important approach of high translational relevance in both therapy (Zuidmeer-Jongejan et al., 2015; Okamoto et al., 2021; Ugajin et al., 2021) and possibly, also prevention.

Allergic reactions to fish are mainly based on type 2 hypersensitivity, thus involving allergen-specific type 2 helper (Th2) cells, which activate B cells to become plasma cells producing IgE-antibodies against fish allergens (Klueber et al., 2019). Fish allergens occur mostly in the fish muscle. The allergen database of the WHO/IUIS allergen nomenclature subcommittee comprises presently 40 fish allergens from 18 different fish species (Matricardi et al., 2016). Most allergens belong to the parvalbumin family, in addition to muscle enolases, aldolases, tropomyosins, triosephosphate isomerases and others. The most potent fish allergen is parvalbumin (Matricardi et al., 2016). This small muscle protein (10–12 kDa) is a calcium-binding protein of high stability towards effects of food processing (Costa et al., 2022). Parvalbumin does not only survive heat treatment, hence causing symptoms upon ingestion of prepared fish, it also can become aerosolized during fish handling, leading to respiratory symptoms upon inhalation. Muscle parvalbumin contents vary considerably in different fish species, which allows to explain the low allergenicity of tuna and swordfish, vs. the high allergenicity of cod and salmon (Kuehn et al., 2010; Mourad and Bahna, 2015; Kobayashi et al., 2016b; Kalic et al., 2019; Pérez-Tavarez et al., 2021). Parvalbumins seem to be also mainly implied in clinical cross-reactivity. Indeed, highly conserved IgE-epitopes occur across the fish species, representing an important reason why fish allergic patient mostly react to various fishes (Kuehn et al., 2014; Hilger et al., 2017). Multiple studies using different technology approaches focused on the identification of B cell epitopes of fish parvalbumins (Perez-Gordo et al., 2013; Carrera et al., 2019; Sharp et al., 2021), as they represent important targets motifs for developing vaccination strategies. Molecular changes can reduce the allergenicity of single fish molecules, which has been successfully shown in a pilot study on epitope-modified mutants of fish parvalbumins (Swoboda et al., 2013). A previous EU-project focused on the subcutaneous application of such a hypoallergenic, low IgE-binding fish allergen variant for the causative treatment of fish allergy (Zuidmeer-Jongejan et al., 2015). The drawback of such approaches is that, if translated into fish farming, genetically modified organisms are created, which cause controversy in human nutrition. Natural, low allergenic fish, such as tuna and ray, are of interest in this context, however they cannot serve the market. Other strategies to reduce the allergenicity of fish parvalbumin, through processing effects inducing protein glycosylation, aggregation or others, perform in the experimental setting (Pérez-Tavarez et al., 2019; Wu et al., 2022), though confirmatory *in-vivo* data are evasive.

A highly promising, innovative, and sustainable approach is to modify the allergenic properties of fish through targeted fish fed in aquaculture. Such hypoallergenic fish from aquaculture hold promise for valuable use in multiple domains. This fish might be used in the frame of fish allergy prevention, by early introduction instead of traditional fishes, to reduce the likelihood for allergic sensitization in high-risk families (Figure 1). Clearly, such hypoallergenic fish will be of relevance for fish-allergic patients, either as a safe alternative to balance their diets or even, as a potentially tolerance-inducing target in safe oral immunotherapy approaches.

### Role of Nutrition is Fish Allergenicity

**FIGURE 2:**
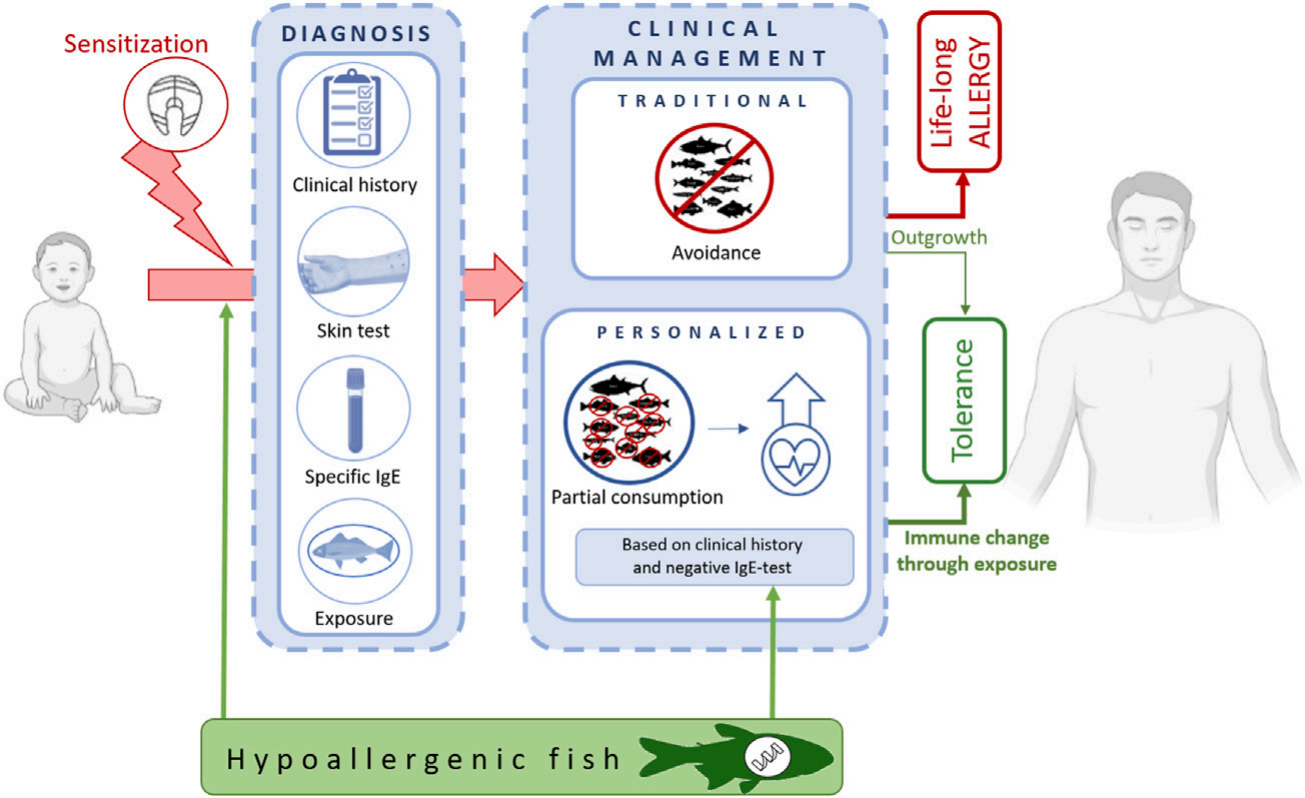
Fish diet supplementation strategy used in gilthead seabream (*Sparus aurata*) and European seabass (*Dicentrarchus labrax*) allergenicity modulation studies. Both species were fed for 3 months, in separate trials, using diets supplemented with different incorporation percentages of EDTA and creatine. EDTA-supplemented diets aimed at inducing the apo-form of the major fish allergen parvalbumin, a calcium-devoid conformation of this protein reported to be less immunoreactive, while the diets supplemented with creatine targeted parvalbumin’s abundance.

Fish is considered nowadays a healthier alternative to protein from other animals. This, together with capture restrictions resulting from wild fish exploitation translates in a worldwide increasing demand for fish and fish products, with aquaculture reaching the XXI century as the fastest-growing animal production sector industry (FAO, 2020). Aquaculture not only alleviates the pressure on marine fish stocks but also allows, in marked contrast to wild-caught fish, for a close monitorization of the entire fish production process, in a controlled environment, ensuring a safe and wholesome aquatic food product for human consumption. Fish nutrition is one of the main aquaculture research areas due to its essential role in promoting fish resilience, welfare, health, and growth, but also in improving the industry’s sustainability and the safety of the final product. Remarkable achievements have been made regarding dietary manipulation and feed supplementation towards enhancing the nutritional value and safety of fish products, e.g., diet enrichment with omega-3 fatty acids and other important nutrients, elimination of toxins, environmental contaminants, and veterinary drug residues (Tacon et al., 2020). Recently fish nutrition has also demonstrated to be a promisor approach to produce a hypoallergenic farmed fish, through the use of fish enriched diets with molecules capable of modulating fish allergenicity by tackling the concentration in muscle or inducing structural modifications in the main fish allergen protein parvalbumin. This was the approach reported in several key studies with gilthead seabream and European seabass (Schrama et al., 2018; De Magalhaes et al., 2020; Schrama et al., 2022), in which authors used creatine or ethylenediamine tetra-acetic acid (EDTA) enriched fish diets to either reduce parvalbumin concentration or induce apo-parvalbumin (a free calcium binding ion structural form of parvalbumin with reduced IgE-binding capacity), respectively (Figure 2). This approach can be considered a highly innovative strategy, given that forefront research is mostly focusing on treating food allergies using immunotherapies, while these studies target a hypoallergenic fish for food allergy prevention. A market study conducted in Portugal, evaluating the consumers’ willingness to pay for a hypoallergenic fish, concluded not only that half the consumers were willing to pay extra for this type of product, but also suggested that the willingness to pay for it was explained by the presence of the same family fish-allergy related pathologies and the fish unique characteristics and quality (Pereira et al., 2020).

The experimental methodology used by authors involves the whole fish production process, from the formulation of diets, growth tests, animal welfare test, the quality and protein characterization of fish muscle, and finally testing *in vitro* the immunological reaction through an IgE-antibody binding test, using sera from patients with a proven fish allergy pathology. To monitor biological and metabolic modifications related to the use of both Creatine and EDTA enriched diets, authors used cutting-edge technologies like Proteomics and Metabolic Fingerprinting by Solid Phase Transmissive Fourier Transform Infrared (FT-IR) Spectroscopy (Schrama et al., 2018; De Magalhaes et al., 2020; Schrama et al., 2022). Proteomics has been the key technology used in many fish nutrition studies over the last decade with proven success in measuring biological effects associated with nutritional factors or a particular dietary treatment. The liver is the target organ in most of these studies due to its role in metabolism regulation and adaptation nutritional changes. Nevertheless, other organs/fluids like muscle, gut/intestine, plasma or mucus have also been successfully used in comparative proteomics studies (de Vareilles et al., 2012a; de Vareilles et al., 2012b; Matos et al., 2013; Schrama et al., 2017; Boonanuntanasarn et al., 2019; Cerqueira et al., 2020).

EDTA is a chelating agent widely present in industrial, cosmetic and food products. Its salt form, calcium disodium EDTA (CAS number: 62-33-9), is an authorized multifunctional food additive (E385) in the European Union (EU), according to Annex II and Annex III of the Regulation (EC) No 1333/2008 on food additives. The maximum levels allowed in several food categories range from 75 to 250 mg/kg, and the acceptable daily intake (ADI) is set to 1.9 mg/kg of body weight (Younes et al., 2018).

Several studies, on different fish species, have demonstrated that calcium-chelating is crucial to the structural preservation of parvalbumin and its IgE-binding capacity (Swoboda et al., 2007; Permyakov et al., 2008; Swoboda et al., 2013; Moraes et al., 2014; Kumeta et al., 2017). Thus, EDTA has been used in several fish allergens-related studies, to induce, *in vitro,* the apo-parvalbumin, i.e., the Ca^2+^-free form of parvalbumin, and explore its reduced IgE-affinity and potential decreased allergenicity. For example, in 1979, Apold & Elsayed used EDTA to induce the uncoupling of the calcium ions from the cod parvalbumin, Gad c 1. The authors reported that this allergen form, devoid of Ca^2+^, showed a IgE-reactivity reduction of around 30% (Apold and Elsayed, 1979). More recently, Mohammadi et al. (2017) showed a significantly decreased IgE-reactivity of the EDTA-treated wolf-herring parvalbumin, compared to the native protein. Additionally, other authors using a different chelating agent, the ethyleneglycoltetraacetic acid (EGTA), achieved similar results and conclusions regarding parvalbumin’s stability and immunoreactivity (Bugajska-Schretter et al., 1998; Tomura et al., 2008; Kobayashi et al., 2016a).

In the aquaculture field, EDTA has been used as fish preservative (Power et al., 1968; Chang et al., 2021) and feed supplement for different fish species, the later targeting heavy metal sequestration (Shalaby, 2007; Gopal et al., 2009; Iranshahi et al., 2011; Abdel-Tawwab et al., 2017) and fish allergenicity modulation (De Magalhaes et al., 2020; Schrama et al., 2022). In these recent studies, the *in vivo* capacity of EDTA to induce a calcium-depleted less reactive parvalbumin was assessed. The authors intended to change the ionic environment of the fish muscle, by using this chelating agent to scavenge for calcium ions, and therefore promote a potentially hypoallergenic fish (reduced content of Ca^2+^-bounded parvalbumin in the muscle). Two important Southern Europe aquaculture fish species, gilthead seabream and European seabass, were fed different diets, specifically formulated for this study, and supplemented with different concentrations of EDTA, for approximately 3 months. A concentration of 3% of EDTA showed a reduction of approximately 50% in gilthead seabream parvalbumin’s IgE-reactivity to cod-allergic patients’ sera, however no changes regarding European seabass were observed with supplementations of up to 4.5% of EDTA. Zootechnical, comparative gel-based proteomics and sensory analyses showed that EDTA supplementation did not affect fish growth, muscle metabolism and fish quality as edible food, respectively, in both species, unless higher concentrations of EDTA were used with negative effects on fish growth performance and feed efficiency. Moreover, to show that this potential hypoallergenic fish was safe for human consumption, the EDTA retained in the fish muscle was assessed by high-performance liquid chromatography (HPLC), and the concentration was below the aforementioned values established by the EU, for the two species (De Magalhaes et al., 2020; Schrama et al., 2022).

Follow-up IgE-reactivity studies using patients’ sera with a confirmed clinical reactivity to these species should be performed to improve and corroborate the reported results, since cases of monosensitivity to specific fish species or single parvalbumin isoforms are common (Kuehn et al., 2011). Enzyme-linked immunosorbent assays (ELISA) with antibodies specific for these species and the purified allergen should also be included in subsequent tests to confirm the reduction in the IgE-binding capacity upon EDTA presence. Lastly, it is important to note that to confirm a reduction in the allergenic potency of this protein more tests should be performed, such as *in vitro* digestibility tests, skin-prick tests, basophil histamine release assays and at the very end double-blind placebo-controlled food challenges (DBPCFC).

Creatine supplementation into fish diets has been investigated in several studies involving different fish species. Growth performance, sprint endurance and allergenicity were analysed in red drum (*Sciaenops ocellatus*) (Burns and Gatlin, 2019), rainbow trout (*Onchorhynchus mykiss*) (McFarlane et al., 2001) and Southern Europe species like gilthead seabream (*Sparus aurata*) (Schrama et al., 2018) and European seabass (*Dicentrarchus labrax*) (Schrama et al., 2022), respectively. In case of the Southern Europe species, creatine was used as a feed supplement aiming to target the expression of the major fish allergen and modulate these species’ allergenicity. Creatine is an important substrate for the energy homeostasis (Schrama et al., 2018), which is essential for all living organisms, including fish, and a well-known supplier for this energy is adenosine triphosphate (ATP) (Borchel et al., 2019). The reversible mechanism of creatine and ATP produce creatine phosphate and adenosine diphosphate (ADP), using the catalysing enzyme creatine kinase (Borchel et al., 2014). Also, a very low amount of creatine/creatine phosphate produces irreversibly creatinine, which is excreted, and therefore creatine needs to be synthesized or ingested through the diet (Borchel et al., 2019). Synthesis of creatine is regulated by the GATM (l-arginine:glycine amidinotransferase) and GAMT (guanidinoacetate-N-methyltransferase) enzymes (Schrama et al., 2022). Studies in rainbow trout confirmed the presence of GATM in muscle tissue (Borchel et al., 2019). Besides this, free and phosphorylated creatine has been found in the fish white muscle (Hunter, 1929), where it also contributes to the contraction/relaxation mechanism of this tissue (Schrama et al., 2018). Additionally, the expression of several proteins present in the muscle, with calcium (Ca^2+^) binding properties, influence the contractile properties of this tissue (Berchtold et al., 2000). Although the creatine metabolism in fish is not fully understood, it seems that the creatine mechanism also influences the calcium homeostasis (Kim et al., 2015). Ingestion of creatine seems to increase Ca^2+^-ATPase, resulting in less available free Ca^2+^ buffering (Kim et al., 2015). A well-known fish muscle Ca^2+^-binding protein is parvalbumin, which is described earlier. Thus, as creatine seems to reduce the calcium content, a decreased expression of calcium binding proteins, such as parvalbumin, might occur, and this was the rationale behind the allergenicity-modulation studies using creatine supplementation.

A control diet was supplemented with three different concentrations of creatine (2, 5 and 8%) and fed both species daily for 3 months, until satiety (Schrama et al., 2018; Schrama et al., 2022). Authors analysed the expression of the allergenic protein parvalbumin, in fish muscle samples, using comparative gel-based proteomics, besides the determinations of fish growth, welfare and quality. Muscle proteome showed a vast number of proteins present in this tissue, including the parvalbumin protein. However, no direct evidence was found that creatine supplementation, with these incorporation percentages, reduced the expression of parvalbumin and consequently allergenicity, neither in gilthead seabream nor in European seabass. Also, zootechnical results showed that Creatine supplementation did not affect fish growth, for both species. Although some differences were found in cortisol concentrations, authors could not link it directly with the experimental diets. Fish quality as edible food was preserved in gilthead seabream (no results were shown for European seabass).

Different studies should be conducted to understand the interaction between supplemented creatine and parvalbumin, including determination of calcium content in fish muscle (as this ion also regulates ATP (Berchtold et al., 2000)). Also, different concentrations of supplementation could be tested, as up to 8% of creatine did not show any negative effect in fish growth or creatine accumulation in muscle (Schrama et al., 2018; Schrama et al., 2022).

## Conclusion

With the rise of food allergy incidence in the world and the absence of a cure, it is imperative to look at innovative strategies to fight this chronic disease. With fish being one of the top nine food allergens and increasingly seen as a source of healthy protein and high nutritional value, the new approach that relies on the modulation of the main fish allergen, with the perspective of allowing allergic patients to still include this nutritionally important food in their diets, is certainly seen as unique and promising, considering the recently published results. Nonetheless, we anticipate a few drawbacks in this new approach as there are limitations in the concentration of molecules like Creatine or EDTA in fish diets as they might negatively interfere with fish feed intake and may also accumulate in fish muscle in concentrations above those legally accepted. On the other hand, the reduction of approximately 50% in gilthead seabream parvalbumin’s IgE-reactivity to cod-allergic patients’ sera, when a concentration of 3% EDTA was added to fish diets, points to a promising strategy. We believe that supplementing fish diets with natural sources like microalgae reported to be rich in compounds with calcium chelating activity, can be a possible strategy. Hence, supplementation of fish feed with microalgal extracts could act both to supply calcium chelators to reduce the fish allergenicity and decrease the need for fish meal as a supply of polyunsaturated fatty acids (PUFA), thus increasing the economic and environmental sustainability of aquaculture.
